# Evaluating remote photoplethysmography: A 10-minute video dataset in uncontrolled lighting

**DOI:** 10.1016/j.dib.2025.111888

**Published:** 2025-07-16

**Authors:** Gonçalo Rodrigues, Nuno M. Garcia

**Affiliations:** Faculty of Sciences of the University of Lisbon (FCUL), Campo Grande 016, 1749-016 Lisboa, Portugal

**Keywords:** Remote Photoplethysmography (rPPG), Remote Heart Rate Estimation, Natural Lighting Conditions, Long-Duration Facial Video

## Abstract

Remote photoplethysmography (rPPG) is a technique that enables the extraction of physiological parameters, such as heart rate, from video recordings in a completely non-contact manner. Although widely studied, rPPG research has been hindered by the scarcity of long-duration and complex video datasets recorded in realistic, everyday scenarios. In this work, we present a dataset comprising 10-minute facial video recordings of 26 participants, along with recorded electrocardiograms (ECG) used as a reference signal. The recordings were acquired using a low-cost RGB webcam and a research-grade ECG monitor, under natural daylight and uncontrolled ambient lighting conditions. Videos were manually synchronized with the ECG and cropped to a 64 × 64 pixel resolution to ensure subject anonymization. While participants were predominantly young Portuguese university students, and Fitzpatrick skin types I–IV are represented, this limits generalizability to broader populations. Each recording includes auxiliary metadata such as temperature, humidity, and illumination levels measured at the beginning and end of the session. The dataset aims to support the development and benchmarking of rPPG algorithms under realistic webcam conditions. Baseline heart rate estimations using standard rPPG methods are also provided.

Specifications Table

**Table:** 

Subject	Computer Sciences
Specific subject area	Long-duration Facial Video and ECG Dataset for Remote Photoplethysmography (rPPG) Heart Rate Estimation
Type of data	Videos (.avi), Data Array (.npy), Table (.xlxs)
Data collection	Facial Videos were captured using a standard Microsoft webcam with 1280 × 720 resolution and recording at 30 frames per second. The original videos were recorded under natural lighting conditions and limited to 600 s (10 min). For each video, three regions were extracted: forehead, left and right cheek, resulting in a total of 3 videos per subject (64 × 64 resolution). Subjects were seated and were asked to keep still and to avoid high amplitude movements. The electrocardiogram was recorded using a BITalino device with a 3-electrode system. Additionally, some environmental and analytical information was retrieved and organized into a table.
Data source location	Portugal, Lisbon
Data accessibility	Repository name: Mendeley Data Data identification number: 10.17632/bx8982xgwt.1 Direct URL to data: https://data.mendeley.com/datasets/bx8982xgwt/1 [1]
Related research article	None

## 1 Value of the Data

• **Enhancing Dataset Diversity:** To the authors knowledge, this is among the longest rPPG datasets available. With 10-minute continuous recordings per subject, this corpus enables research into temporal robustness, including signal drift, cumulative noise, and model degradation introduced by spontaneous environmental and physiological changes, scenarios that are difficult to study using short-segment corpora. This helps developing temporal robust models that effectively identify the underlying signal instead of relying on short time patterns.
• **Challenging Real-World Conditions:** Unlike many datasets, videos were recorded under natural daylight with up to 800 lx variation using a low-cost webcam. This introduces realistic noise, making the dataset closer to everyday conditions.
• **Promotes Privacy-Preserving Model Development:** Low-resolution (64 × 64 pixels) anonymized facial ROIs allow for training and evaluating models that respect user privacy, an increasingly important constraint in real-world deployment.
• **Quantitative Baseline for Benchmarking:** The dataset was tested using 3 of the most employed rPPG estimation models.
• **Encourages Open, Reproducible Research:** The dataset is freely available, well-documented, and unconstrained in terms of processing methods, fostering transparent benchmarking and fair evaluation across different rPPG approaches.

## 2 Background

Remote photoplethysmography (rPPG) is a non-contact technique for estimating physiological parameters such as heart rate by analyzing subtle color variations in facial skin [2]. The ability to measure these changes using standard cameras has led to growing interest in rPPG for applications in healthcare and other relevant areas. While rPPG has shown promise as a non-contact method for heart rate estimation, its performance remains highly sensitive to variations in illumination, motion, and individual differences. Many existing datasets are limited in duration and collected under controlled lighting conditions, which do not adequately represent real-world scenarios. This dataset was designed to address these limitations by providing long-duration (10-minute) facial video recordings captured under natural lighting conditions. The goal was to allow researchers to study the long-term stability of rPPG signals and investigate the impact of natural illumination on heart rate estimation. Additionally, the inclusion of ECG as a ground-truth reference enhances the dataset’s value for validating different rPPG methods. This dataset offers a benchmark for evaluating rPPG algorithms in challenging real-world settings. The aim of making this dataset publicly available is to facilitate further research into improving the robustness and applicability of rPPG-based physiological monitoring.

## 3 Data Description

This dataset consists of facial video recordings designed to support research in remote photoplethysmography, facilitating the development of innovative approaches for heart rate measurement from videos. It includes data from 26 healthy Portuguese university students (12 male, 14 female) with a mean age of 22.5 ± 1.2 years. Although a total of 27 subjects were originally recorded, one participant (Subject_4) was excluded from the final dataset due to significant artifacts in both video and ECG data. Each subject has three 10-minute videos (600 s each), synchronized ECG signals, and analytical data. The dataset is structured as follows:

• **Subject-specific folders:** Each subject has a dedicated folder (e.g., *Subject_1, Subject_2*, etc.).
  ○ **Videos:** Three separate videos per subject, each corresponding to a specific region of interest (ROI):
    ■ Forehead
    ■ Cheek1 (Right Cheek)
    ■ Cheek2 (Left Cheek)
    ■ Videos are stored in “.avi” format with a resolution of 64 × 64 pixels and are named following the format *Subject_X_ROI.avi* (e.g., *Subject_1_Forehead.avi*)
  ○ **ECG signals:** Each subject’s corresponding ECG signal is stored as a “.npy” file, labelled *Subject_X_ECG.npy* (e.g., *Subject_1_ECG.npy*), making it easily readable using Python’s NumPy library or similar tools.
• **Analytical and environmental data:**
  ○ An Excel file (“.xlsx”) contains additional metadata for each subject, including:
    ■ Demographic information (age, sex, height, etc.)
    ■ Environmental conditions (temperature, humidity, recording time, etc.)

## 4 Experimental Design, Materials and Methods

### 4.1 Subject selection

The selection of subjects was made with certain conditions in mind. First, the aim was to achieve a balance between male and female participants to improve the generalizability of the dataset. Second was to include individuals from various ethnic backgrounds and with different skin tones. To achieve this, the Fitzpatrick scale was used [3], which assesses skin tone based on visual observation and the subject's susceptibility to sunburn, a factor that was individually discussed with each participant and determined by two researchers. The resulting distribution of skin tones was not as diverse as desired, which limits the broadness of the dataset (*I* = 2, II = 17, III = 4, IV = 3, V & VI = 0).

Additionally, efforts were made to maintain a relatively narrow age range, with a minimum age of 18 to ease ethical requirements. While this reduces the generalizability of the dataset in terms of age, it facilitates the analysis of other data factors by decreasing variability and increasing homogeneity. Beyond these imposed conditions, there was no target population, as the intention was to avoid being bound by the socio-psychological or physiological characteristics of participants. If the age criterion was met, individuals were eligible to participate in the study. The goal was to obtain a randomized sample.

### 4.2 Retrieved information

Each participant provided a facial video recording accompanied by an electrocardiogram (ECG) signal, which serves as the ground truth. These two components form the core of the dataset. In addition to video footage, various participant characteristics were collected to enhance the dataset’s research value.

• **Demographics:** Age, sex, height, weight, and Fitzpatrick skin tone classification to ensure diversity and balanced representation.
• **Health Factors Affecting Heart Rate** [4]**:**
  ○ Presence of medical conditions or diseases.
  ○ Regular physical activity, defined as at least 150 min of moderate-intensity exercise per week, according to WHO guidelines [5].
  ○ Recent consumption of alcohol within 12 h prior to recording.
  ○ Smoking status.
• **Environmental Data**
  ○ Lightning level measured at the start and end of the recording.
  ○ Ambient conditions such as temperature and humidity.
  ○ Recording metadata: date and time of recording.
• **Factors Affecting Estimation** such as skin obstruction. The presence of makeup, sunscreen, glasses or hair were noted due to their potential impact.

### 4.3 Equipment

The following equipment was used for data collection:

• **Video Recording:** Microsoft webcam (1280 × 720 resolution, 30 fps), using OBS Studio as recording software.
• **Electrocardiogram (ECG):** BITalino with a 3-electrode system (1000 Hz, ±1.5 mV) and OpenSignals software, using disposable Ag/AgCl foam electrodes with semi-liquid gel.
• **Ambient Conditions:** Temperature and humidity measured with a Sensibo Air Pro.
• **Illumination Levels:** Measured using a YFE Digital Light Meter (model YF-1065).

All remaining participant characteristics were assessed through questionnaires or direct observation.

### 4.4 Setup

**Fig. 1:**
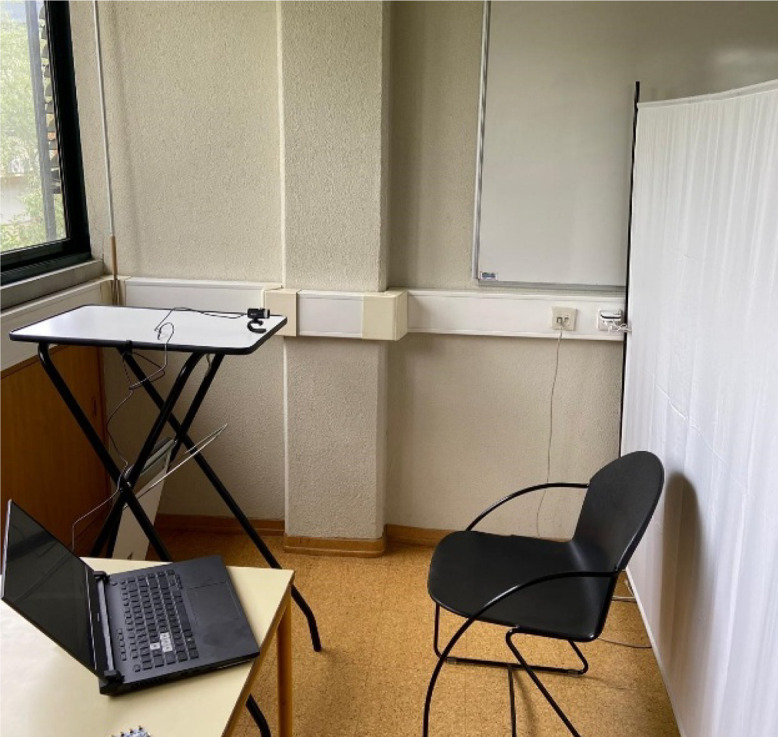
Setup for the acquisition.

The recordings were conducted in a room with only natural light. Subjects were positioned approximately 2 m from the window. Recordings took place between 10 a.m. and 4 pm. to ensure sufficient lighting, although on some days, illumination was considerably lower due to cloudy weather. The camera was positioned roughly 1 m from the subjects' faces and at approximately the same height. Recordings were done against a white background, with participants seated. The device used to measure room conditions was placed to the right of the subjects, and illumination measurements were taken twice, at the start and end of the recording, with the light sensor positioned in front of the subjects' faces. An image of the setup can be seen in Fig. 1.

The electrodes were positioned on the wrists and the left hip. The ECG setup also included an input button that, by generating a pulse in the recorded signal, allowed for synchronization between the video and the biomedical signal. The ECG recording was initiated using a manual trigger pulse, while video capture was started manually by the operator immediately thereafter. As the trigger signal was only present in the ECG stream and not embedded in the video, exact synchronization between the two modalities was not enforced via hardware. Based on typical human reaction time variability (200–300 ms) and additional software startup latency, we estimate a conservative synchronization uncertainty of up to ±500 ms. This delay may introduce a fixed offset between the physiological signals but is unlikely to meaningfully affect applications that operate on multi-second time windows such as average heart rate estimation, which is the primary application intended for this dataset. For applications requiring sub-second timing accuracy (e.g., inter-beat interval analysis or pulse transit time estimation), users should interpret timing relationships with caution.

### 4.5 Acquisition protocol

The data acquisition followed a structured protocol:

1. Upon arrival, participants were seated and allowed a short relaxation period to stabilize heart rate and reduce variability (approximately 5–10 min, during which Steps 2 to 5 were completed).
2. Participants received an informational leaflet outlining the experiment and data usage. They were then asked to sign an informed consent form in accordance with ethical guidelines. Any questions were addressed before proceeding.
3. A brief questionnaire was administered to collect data regarding demographics and other health factors that could influence the heart rate.
4. Electrodes were placed on the subject’s wrists and left hip, and the ECG recording system was prepared.
5. Environmental parameters—temperature, humidity, date, and time—were recorded. An initial illumination measurement was also taken using a digital lux meter.
6. Data acquisition proceeded as follows:
  ○ The ECG recording was started first to ensure signal integrity.
  ○ The video recording was initiated simultaneously by the operator with a manual trigger input on the ECG system for synchronization.
  ○ The session lasted 10 min, during which participants were instructed to remain still, facing the camera.
  ○ To minimize distractions, participants were left alone in the room during the recording.
  ○ Upon completion, the integrity of both ECG and video recordings was verified, and a final illumination reading was taken.
7. All data were saved and organized for further processing.

### 4.6 Data processing

All data processing was carried out in Google Colab using Python. The used code is accessible in this repository.

**1. Video Recording Processing**

All the video processing was mainly carried out using the OpenCV library for Python. The primary objective during this processing was to maintain the anonymity of the participants to comply with the ethical considerations. This was done by splitting the video into three regions of interest, which avoids the presence of any traits that might enable the identifying of the participants such as the nose, mouth and eyes.

For face detection and tracking, Mediapipe Face Mesh was employed. It is a machine learning approach developed by Google that uses a series of models to detect face landmarks and facial expressions in images and videos [6]. One model detects faces and a second model locates landmarks on the detected faces. It shows consistently on the literature to outperform other means of face tracking and therefore was employed to perform this task [7].

**Fig. 2:**
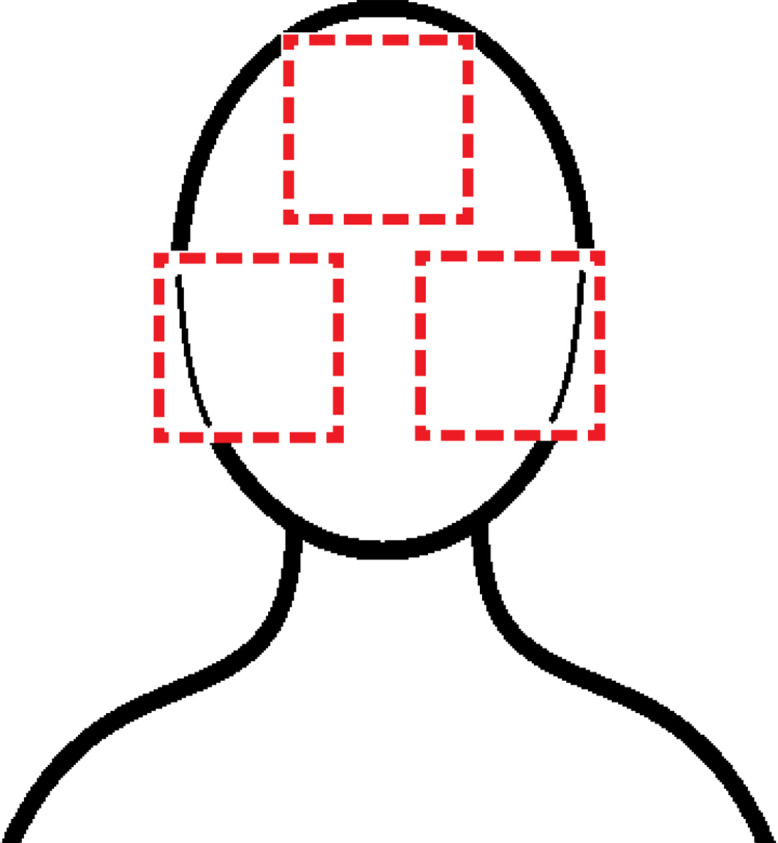
Schematic of ROI cropping.

The regions of interest were defined as the forehead and both cheeks, as these areas are not only the most used for rPPG tasks according to the literature but also yield the best results due to being large flat areas with high vascularization and low skin thickness, enhancing the signal to noise ratio [8]. Selecting these areas simultaneously allow for the anonymization of the subjects by excluding the eyes, nose and mouth. Hence, three feature points were identified in the face, corresponding to central points within each one of the regions. Based on the selected landmarks, bounding boxes were created around them to delimit the Regions of Interest. A schematic example of the ROI cropping is provided in Fig. 2. Subsequently, three separate videos were reconstructed, one for each of the regions. These videos were also limited to a 10-minute duration. To ensure correct anonymization, all resulting videos were manually inspected by the authors to confirm that no personally identifying elements remained visible.

The original video had dimensions of 1280 × 720, while the resulting have 64 × 64, each one featuring the forehead or one of the cheeks. The rationale behind the 64 × 64 resolution was simply that it was empirically determined to cover the entire defined region of interest without exceeding it, avoiding the presence of identifiable features. While this can raise concerns about decreasing the signal to noise ratio, the effect is expected to be minimal when reducing the region size to these dimensions, as shown previously [9]. Furthermore, utilizing a larger ROI is also not linked to better performance, as adding non-skin pixels to the region of interest is also detrimental to the signal to noise ratio [9]. For instance, using a combination of the Cheeks and Forehead has been shown to outperform the whole face as a region of interest [8].

Occasionally, the face tracking model was not able to identify faces and therefore regions. This was easily fixed by assigning the location of regions from the previous frame to the current frame in case one could not be located, which overcame this limitation. This event was observed approximately 15 to 20 times. The flagged frames were stored, and the corresponding video segments were manually reviewed by the researchers. Most of these occurrences did not result from video degradation and appeared to be random. One instance was attributed to the camera being out of focus (Subject_13), while another was caused by a participant coughing and raising her hand to her mouth (last 20 s of Subject_21 recording). These events were brief, each lasting no more than three seconds, and collectively accounted for <0.5 % of the total duration of each video. As such, they are not expected to significantly impact the overall quality of the recordings.

**2. Electrocardiogram Signal Processing**

This data recording originated two signals: the ECG signal and the Input signal produced by the button. During processing, they were firstly imported and separated into the two different source signals. After that, the synchronization step was done by removing the initial part of the ECG signal before the input pulse, which signalizes the start of the facial recording. The remaining signal was also limited to 10 min of length.

**3. Analytical Data**

All the remaining data corresponding to each subject, was manually organized into a table, which is shared with the dataset in an Excel sheet. All the units of measurement are provided there.

### 4.7 Subject exclusion criteria

One subject (Subject_4) was excluded due to severe tracking and ECG quality issues. The face-tracking algorithm flagged multiple periods of failure based on non-tracked frames. Manual video inspection confirmed persistent large-amplitude head movements throughout the recording. Simultaneously, the ECG signal exhibited multiple non-physiological peaks, rendering it unreliable for use as ground truth. This exclusion was made prior to dataset release and did not affect the processing pipeline for other subjects.

### 4.8 Baseline benchmark evaluation

To support the utility of the presented dataset as a benchmark for remote photoplethysmography (rPPG) development, a set of baseline heart rate (HR) estimations was performed using four classical methods:

• GREEN, by Verkruysse et al., 2008 [2]
• ICA, by Poh et al., 2010 [10]
• CHROM, by de Haan and Jeanne, 2013 [11]
• POS, by Wang et al., 2017 [12]

All algorithms were applied using the common processing pipeline provided by the rPPG-Toolbox [13] to ensure comparability. The ROI used is composed of the forehead and both cheeks. Signals were segmented into non-overlapping 30-second windows.

Performance was evaluated using mean absolute error (MAE), mean absolute percentage error (MAPE), root mean square error (RMSE), and Pearson correlation coefficient (Pcc) between the estimated and ground-truth ECG heart rate. The results are summarized in Table 1. These results are not intended as a performance ranking, but rather to provide an indicative baseline for future comparisons.

**Table 1:** Heart rate estmiation baseline performance on four rPPG methods.

Method	MAE (bpm)	MAPE ( %)	RMSE (bpm)	Pcc
GREEN [2]	7.57 ± 0.66	9.13 ± 0.82	16.79 ± 6.55	0.49 ± 0.04
ICA [10]	7.61 ± 0.42	9.06 ± 0.48	12.31 ± 3.87	0.50 ± 0.04
CHROM [11]	10.05 ± 0.46	13.12 ± 0.66	14.56 ± 4.48	0.24 ± 0.04
POS [12]	7.59 ± 0.46	9.82 ± 0.64	12.88 ± 4.89	0.43 ± 0.04

## Limitations

The study faced several limitations.

• Skin tone classification, based on the Fitzpatrick scale, can be subjective and evaluator dependent. Although two researchers assessed skin tones to reduce bias, some error may persist.
• Recruitment was passive, using emails and flyers, which limited ethnic diversity and resulted in only 26 participants. This led to a narrower skin tone range, which may affect signal quality, as skin tone has been shown to influence it.
• Synchronization between video and ECG data relied on a manual trigger pulse, introducing a human reaction delay of up to 500 ms. While acceptable for average heart rate estimation, this could impact applications needing sub-second precision.
• The dataset also constrains users to 64 × 64 regions of interest, potentially risking the loss of signal information.
• Illumination was measured only at the start and end of each session due to hardware constraints, limiting insight into lighting fluctuations during recordings.
• Additionally, face tracking failed in approximately 15–20 brief instances. Most were random, though two had clear causes: camera defocus (Subject_13) and a participant’s hand obstruction (Subject_21). While these issues had minimal overall impact, they are acknowledged as minor limitations of the dataset.

## Ethics Statement

This study was approved by the Ethics Committee of the Faculty of Sciences of the University of Lisbon under the reference CEC/2/2024 and was conducted in accordance with the ethical principles outlined in the Declaration of Helsinki.

All participants provided written informed consent prior to their involvement in the study. The consent process included clear explanations of the study’s purpose, procedures, data handling, and participants’ rights, including the right to withdraw at any time without consequences.

Participants were also informed about how their data would be used, stored, and shared, and their privacy rights were fully respected. Personally identifiable information was not collected, and all data were anonymized prior to publication.

## CRediT Author Statement

**Gonçalo Rodrigues:** Methodology, Software, Investigation, Data Curation, Writing – Original Draft. **Nuno M. Garcia:** Conceptualization, Resources, Writing - Review & Editing, Supervision, Funding acquisition.

## Declaration of Generative AI and AI-assisted Technologies in the Writing Process

During the preparation of this work the author(s) used OpenAI ChatGPT to check for misspelling errors and text clarity. After using this tool/service, the author(s) reviewed and edited the content as needed and take(s) full responsibility for the content of the published article.

## Data Availability

Mendeley DatarPPG-10 (Original data).
